# Off-site primary percutaneous coronary intervention in a new centre is safe: comparing clinical outcomes with a hospital with surgical backup

**DOI:** 10.1007/s12471-016-0872-0

**Published:** 2016-09-05

**Authors:** K. H. A. J. Koolen, K. A. Mol, B. M. Rahel, F. Eerens, S. Aydin, R. P. T. Troquay, L. Janssen, W. A. L. Tonino, J. G. Meeder

**Affiliations:** 1 Department of Cardiology, VieCuri Medical Centre, Venlo, The Netherlands; 2 Department of Cardiology, Catharina Hospital, Eindhoven, The Netherlands

**Keywords:** Primary, Percutaneous coronary intervention, Off-site, Outcomes, Coronary disease

## Abstract

**Objectives:**

To evaluate the procedural and clinical outcomes of a new primary percutaneous coronary intervention (PPCI) centre without surgical back-up (off-site PCI) and to investigate whether these results are comparable with a high volume on-site PCI centre in the Netherlands.

**Background:**

Controversy remains about the safety and efficacy of PPCI in off-site PCI centres.

**Methods:**

We retrospectively analysed clinical and procedural data as well as 6‑month follow-up of 226 patients diagnosed with ST-elevated myocardial infarction (STEMI) who underwent PPCI at VieCuri Medical Centre Venlo and 115 STEMI patients who underwent PPCI at Catharina Hospital Eindhoven.

**Results:**

PPCI patients in VieCuri Medical Centre had similar procedural and clinical outcomes to those in Catharina Hospital. Overall there were no significant differences. The occurrence of procedural complications was low in both groups (8.4 % VieCuri vs. 12.3 % Catharina Hospital). In the VieCuri group there was one procedural-related death. No patients in either group needed emergency surgery. At 30 days, 17 (7.9 %) patients in the VieCuri group and 9 (8.1 %) in the Catharina Hospital group had a major adverse cardiac event.

**Conclusion:**

Performing PPCI in an off-site PCI centre is safe and effective. The study results show that the procedural and clinical outcomes of an off-site PPCI centre are comparable with an on-site high-volume PPCI centre.

## Introduction

Primary percutaneous coronary intervention (PPCI) at hospitals without surgical back-up (off-site PCI) has been frequently investigated and debated. PPCI is an effective treatment in acute coronary syndrome (ACS) and superior to thrombolytic therapy [1–3]. The knowledge that a decrease in time to reperfusion leads to decreased infarct size and incidence of major adverse cardiac events (MACE), contributed to the rise of off-site PCI centres [2, 4–6].

Introduction of PCI at off-site hospitals has been a gradual process in the Netherlands and implementation is strictly regulated [7]. Numerous studies have reported no difference in safety and effectiveness of PCI between off-site PCI centres and medical centres with surgical back-up (on-site) [3, 8–12], including two large Dutch studies [13, 14]. The need for bail-out surgery after on-site PCI has dropped dramatically in the past decades, from 6.6 % in the initial years to 0.3–0.6 % currently [15, 16]. The mortality rates for patients requiring emergency surgery are the same in off-site and on-site PPCI centres [17, 18]. The European Society of Cardiology (ESC) recommends (Ib) PPCI to be performed by experienced operators in a 24-hour/7-day service [6]. No distinction is made between on-site and off-site centres while, according to the American College of Cardiology (ACC)/American Heart Association (AHA) guidelines, PPCI at off-site centres is a class IIa indication [19]. Both the ESC and ACC recommend that operators performing PCI for ACS should have an annual volume of at least 75 procedures at institutions performing at least 400 PCIs per year [19, 20].

The aim of this study is to investigate whether the results at the VieCuri Medical Centre Venlo are comparable with a high-volume on-site PCI centre, in this case Catharina Hospital Eindhoven. We assume the procedural and clinical outcomes are similar for off-site PPCI compared with on-site PPCI.

## Methods

This study is a two-centre, retrospective cohort study. In the period from 1 September 2012 to 1 September 2013, 122 patients in the VieCuri Medical Centre/Laurentius Hospital Roermond area received PPCI in Catharina Hospital. From September 2013, patients from Laurentius Hospital and VieCuri were treated at VieCuri Medical Centre.

All STEMI patients who were signed up for PPCI were included. Patients with an out-of-hospital cardiac arrest were excluded. VieCuri is an intermediate-volume hospital which started PPCI in September 2013. Laurentius Hospital is an intermediate-volume hospital without PCI facilities. Before September 2013 patients from VieCuri and Laurentius Hospital who needed PPCI were transported to Catharina Hospital.

### PCI procedure

PPCI is limited to the culprit vessel with the exception of patients presenting with cardiogenic shock or persistent ischaemia after PCI of the presumed culprit lesion [20]. The choice for drug-eluting stent (DES) versus bare metal stent and the use of an intra-aortic balloon pump or glycoprotein IIb/IIIa inhibitors, was left to the discretion of the interventional cardiologist.

### Data collection and outcome measures

Patient characteristics, PCI characteristics, complications and follow-up data were retrospectively found in the medical records. Missing data from PPCI patients were acquired by calling general practitioners. Foreign patients, transferred to different countries for rehabilitation, were considered as lost to follow-up.

The primary outcomes of this study were complications during the procedure and the incidence of a major adverse cardiac event (MACE) at 30 days and 6 months. Only procedural complications were registered and no complications as a result of the myocardial infarction itself. The combined endpoint MACE consists of death, myocardial infarction and revascularisation (target-lesion, target-vessel or non-target-vessel). Secondary outcomes are the incidence of major adverse cardiovascular and cerebral events (MACCE) and consist of death of any cause, myocardial infarction, revascularisation (target-lesion, target-vessel or non-target-vessel), emergency or semi-elective coronary artery bypass graft (CABG), occurrence of cerebral vascular accidents, probable or definite stent thrombosis, TIMI major and minor bleeding [21] and the need for transfusion. Emergency CABG was defined as CABG performed within 24 hours after PCI for a procedural complication. Secondary outcomes were analysed with a maximum follow-up period of 6 months.

Primary outcome registration was accomplished by definitions from the Academic Research Consortium (ARC) [22]. All deaths are considered cardiac unless an unequivocal noncardiac cause could be established. Re-PCI was defined following ARC definitions with target lesion revascularisation, target vessel revascularisation and non-target vessel revascularisation. Target lesion revascularisation before 30 days is considered to be a safety endpoint, because this time is too short for fibrointimal hyperplasia [22, 23]. Stent thrombosis was classified as definite, probable and possible and timing of the stent thrombosis as acute, subacute or late [22].

### Data analysis

Data were collected and analysed by an independent investigator in SPSS version 22. Descriptive statistics were used to calculate frequencies and means. The independent sample T‑test and the Mann-Whitney test were used to compare means. Chi-square or Fisher’s exact test were used to compare the VieCuri data with those of Catharina Hospital.

## Results

A total of 122 and 237 PPCIs in patients diagnosed with STEMI were conducted in Catharina Hospital and VieCuri, respectively. This consists of 115 and 226 patients. There were 74 patients from Laurentius Hospital who underwent PPCI in VieCuri Medical Centre. In the Catharina Hospital group, 21 patients (18.3 %) were first admitted to VieCuri before transportation to Catharina Hospital for PPCI.

### Characteristics

Baseline characteristics and prescribed medication are shown in Table 1. The patient groups were clinically well balanced for all risk factors; however, there were significantly more patients with Killip class II in the VieCuri group and the TIMI risk score was significantly higher compared with the Catharina Hospital group. There was a significant difference in the prescription of aspirin, beta blockers, aldosterone antagonist and proton pump inhibitors between VieCuri and Catharina Hospital. Several patients did not have dual antiplatelet therapy because either no stent had been placed or CABG was necessitated.

**Table 1:** Baseline characteristics

Characteristics	VieCuri (*n* = 226)	(*n* = 115)	*p*
Mean age, years (SD) Male gender, *n* (%) Mean BMI (SD) Diabetes, *n* (%) Hypertension, *n* (%) Hypercholesterolaemia, *n* (%) Smoker, *n* (%) – Unknown, *n* (%) Family history of CAD, *n* (%) – Unknown, *n* (%) Peripheral vessel disease, *n* (%) Previous MI, *n* (%) Previous PCI, *n* (%) Previous CABG, *n* (%) Previous stroke/TIA, *n* (%) Renal disease, *n* (%) Metastatic cancer, *n* (%) LVEF <0.40, *n* (%) Mean CK max u/g (SD) Killip class 1 Killip class II Killip class III Killip class IV TIMI risk score (SD)	62.83 (12.34) 165 (73.0 %) 27.03 (4.10) 30 (13.6 %) 91 (41.2 %) 66 (29.9 %) 106 (48.0 %) 24 (10.9 %) 97 (43.9 %) 44 (19.9 %) 22 (10.0 %) 30 (13.6 %) 26 (11.8 %) 7 (3.2 %) 17 (7.7 %) 16 (7.2 %) 5 (2.3 %) 17 (8.3 %) 1363 (1709) 148 (81.8 %) 15 (8.3 %) 7 (3.9 %) 11 (6.1 %) 3.06 (2.55)	62.29 (13.40) 81 (70.4 %) 26.92 (3.93) 9 (8.0 %) 44 (38.9 %) 41 (36.3 %) 46 (40.4 %) 10 (8.8 %) 53 (46.5 %) 22 (19.3 %) 14 (12.4 %) 19 (16.5 %) 16 (13.9 %) 4 (3.5 %) 7 (6.2 %) 6 (5.3 %) 4 (3.5 %) 7 (7.1 %) 1385 (1450) 112 (92.6 %) 3 (2.5 %) 2 (1.7 %) 4 (3.3 %) 2.23 (1.95)	0.708 0.616 0.946 0.131 0.693 0.234 0.122 – 0.900 – 0.497 0.468 0.572 1.000 0.616 0.507 0.494 0.720 0.882 0.008^*^ 0.037^*^ 0.323 0.418 0.047^*^
*Medication* ^a^			
Aspirin, *n* (%) Clopidogrel, (%) Prasugrel, *n *(%) Ticagrelor, *n* (%) Vitamin K antagonist, *n* (%) ACE inhibitor, *n* (%) Angiotensin-II inhibitor, *n* (%) Beta blocker, *n* (%) Statin, *n* (%) Nitrate, *n* (%) Calcium channel blockers, *n* (%) Diuretics, *n* (%) Aldosterone antagonist, *n* (%) Proton pump inhibitor, *n* (%)	208 (96.7 %) 54 (25.1 %) 85 (39.5 %) 73 (34.0 %) 11 (5.1 %) 162 (76.1 %) 46 (21.6 %) 199 (92.6 %) 213 (99.1 %) 99 (46.0 %) 17 (7.9 %) 25 (11.6 %) 14 (6.5 %) 277 (82.3 %)	104 (91.2 %) 29 (25.4 %) 37 (32.5 %) 47 (41.2 %) 10 (8.8 %) 93 (81.6 %) 16 (14.0 %) 112 (98.2 %) 114 (99.1 %) 57 (50.0 %) 8 (7.0 %) 20 (17.5 %) 17 (14.9 %) 105 (92.1 %)	0.031^*^ 0.949 0.206 0.192 0.197 0.251 0.096 0.031^*^ 0.546 0.494 0.772 0.137 0.013^*^ 0.016^*^

*BMI* body mass index, *CAD* coronary artery disease, *MI* myocardial infarction, *PCI* percutaneous coronary intervention, *CABG* coronary artery bypass graft, *TIA* transient ischaemic attack, *LVEF* left ventricular ejection fraction,* CK* creatine kinase*, ACE* angiotensin-converting-enzyme, *DES* drug-eluting stent

^*^Significant difference

^a^Prescribed medication at discharge

### PCI specifications

**Fig. 1:**
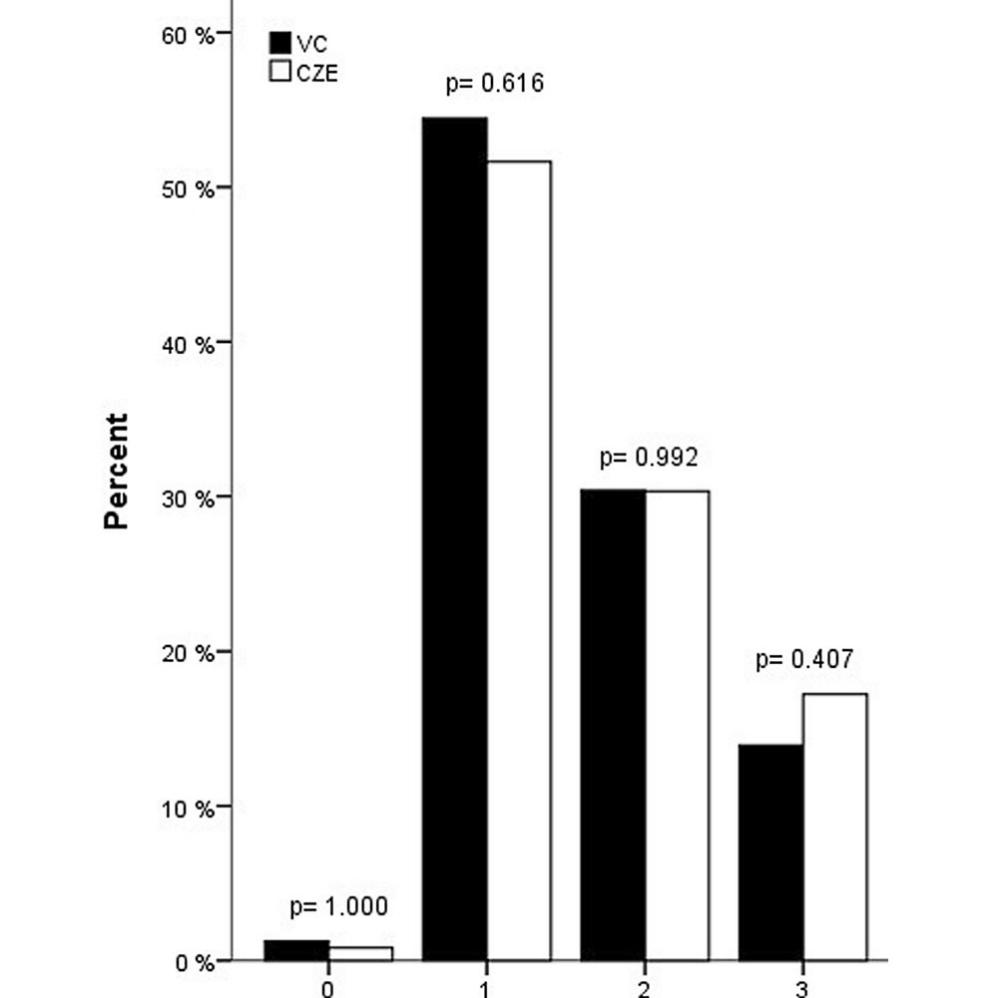
Number of vessel disease. (*VC* VieCuri Medical Centre Venlo, *CZE* Catharina Hospital Eindhoven)

**Fig. 2:**
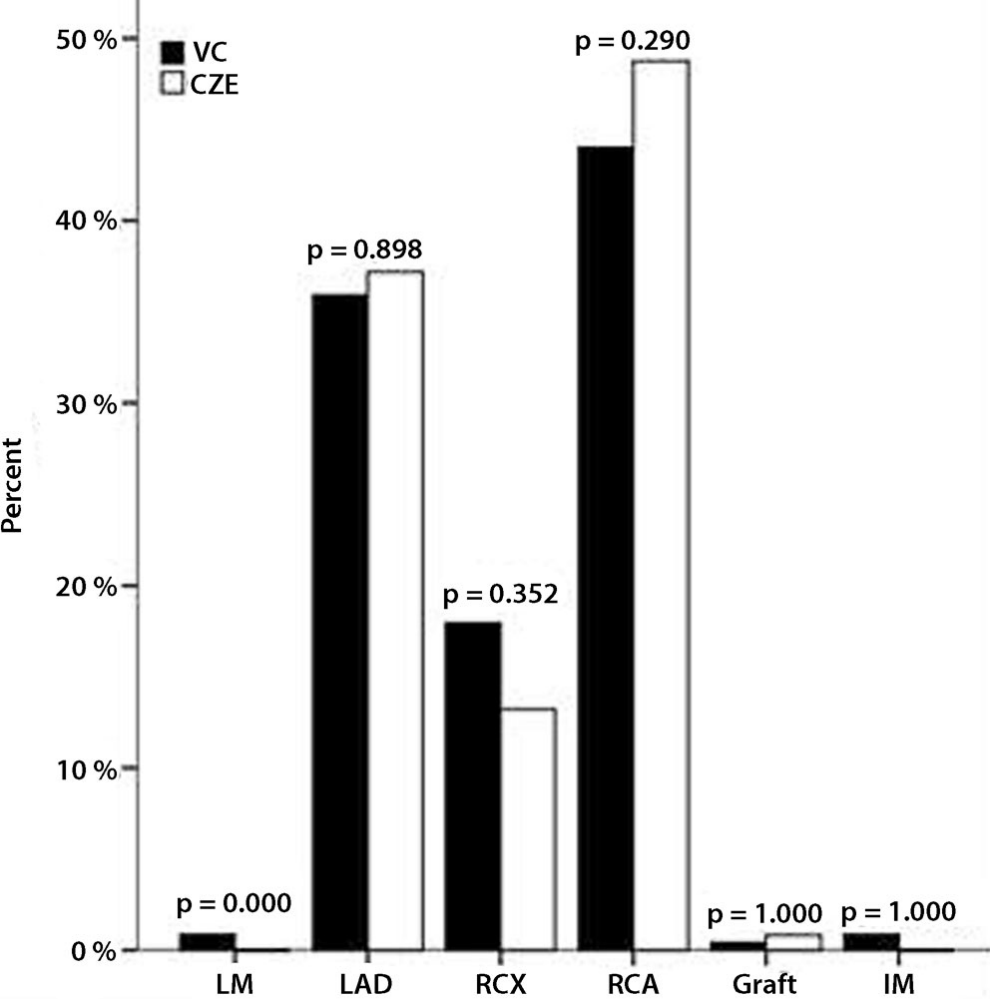
Culprit vessel. (*VC* VieCuri Medical Centre Venlo, *CZE* Catharina Hospital Eindhoven, *LM* left main, *LAD* left anterior descending, *RCX* right circumflex, *RCA* right coronary artery)

**Fig. 3:**
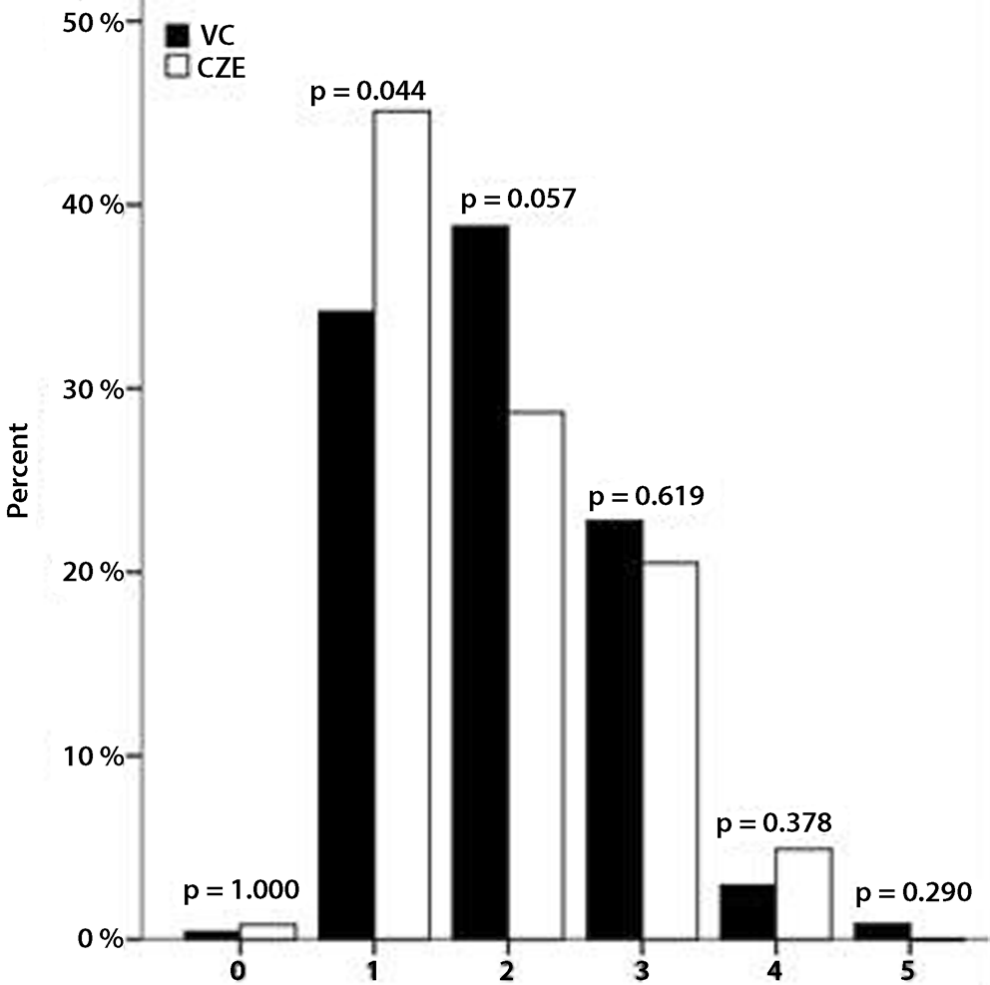
Number of lesions. (*VC* VieCuri Medical Centre Venlo, *CZE* Catharina Hospital Eindhoven)

**Fig. 4:**
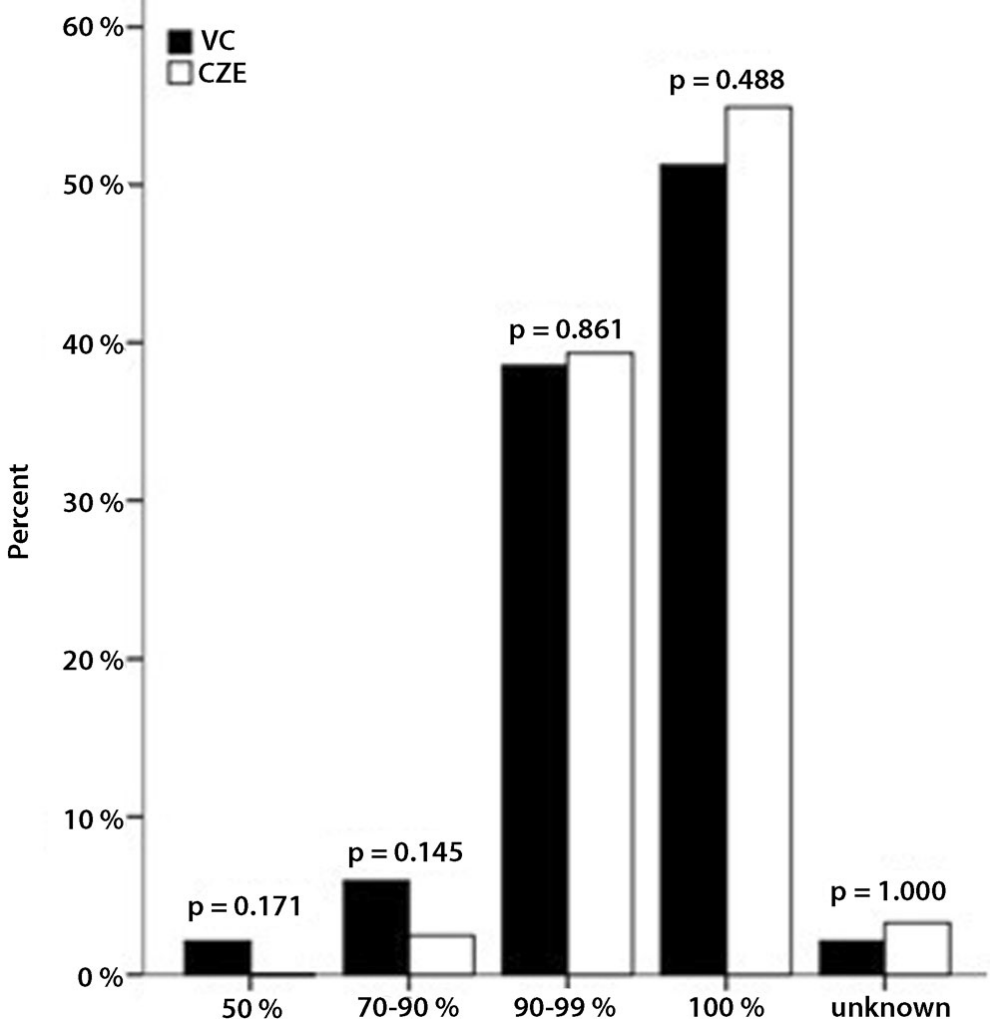
Diameter stenosis as percentage. (*VC* VieCuri Medical Centre Venlo, *CZE* Catharina Hospital Eindhoven)

Fig. 1, 2, 3 and 4 show the PCI specifications. Most patients had one-vessel disease (54.4 %) and the right coronary artery was the most common culprit vessel (44.0 %), which is shown in Fig. 1 and 2, respectively. Data from Catharina Hospital show the same distribution. There was a significant difference in the number of patients with one lesion (Fig. 3), which was higher in the Catharina Hospital group. In most patients, the diameter of the stenosis was 100 % (Fig. 4).

Table 2 shows the PCI specifications of both VieCuri and Catharina Hospital. In 215 of the 237 PPCIs a stent was placed in one of the coronary arteries. In 80.7 % this was a DES. There were 22 procedures in which no stent was inserted. In 6 cases stent placement was not successful. In 4 cases the operator decided, after spontaneous reperfusion, not to place a stent due to multivessel disease or stenosis of the left main coronary artery needing CABG. In both groups only a few radial procedures were performed, because this was up-coming at that period. The time from first medical contact to start of PCI (system delay) was significantly longer in the Catharina Hospital group.

**Table 2:** Procedural specifications

Procedural specifications^a^	VieCuri (*n *= 237)	Catharina (*n* = 122)	*p*
Procedures without stent placing – Unsuccessful, *n* (%) – Multivessel disease necessitating CABG, *n* (%) – Only balloon dilatation Radial procedure, *n* (%) DES, *n* (%) Stents/PCI Mean total stent length, mm (SD) Time FMC to start PCI, min (SD)	22 (9.3 %) 6 (2.5 %) 4 (1.7 %) 12 (5.1 %) 8 (3.4 %) 268 (80.7 %) 1.53 19.67 (6.12) 76 (32)	8 (6.9 %) 4 (3.3 %) 1 (0.8 %) 3 (2.5 %) 5 (4.1 %) 135 (90.0 %) 1.28 16.05 (6.01) 101 (91)	0.377 0.242 0.711 0.231 0.769 – – 0.445 0.000^*^
Total complications, *n* Procedures with complications, *n* (%) Unsuccessful with complication, *n* (%) Acute vessel closure, *n* (%) Coronary dissection, *n* (%) Femoral/radial dissection, *n* (%) Stent thrombosis, *n* (%) No-reflow, *n* (%) MI, *n* (%) Cardiac arrest, *n* (%) – Ventricular fibrillation, *n* (%) Coronary perforation, *n* (%) Cardiac tamponade, *n* (%) Haemodynamic instability, *n* (%) CVA, *n* (%) TIMI major bleeding, *n* (%) TIMI minor bleeding, *n* (%) Pseudoaneurysm^b^, *n* (%) Transfusion, *n* (%) Need for cardiac surgery, *n* (%) Procedure related death, *n* (%)	28 20 (8.4 %) 1 (0.4 %) 0 (0.0 %) 4 (1.7 %) 0 (0.0 %) 4 (1.7 %) 0 (0.0 %) 2 (0.8 %) 5 (2.1 %) 4 (1.7 %) 1 (0.4 %) 2 (0.8 %) 3 (1.3 %) 0 (0.0 %) 0 (0.0 %) 1 (0.4 %) 3 (1.3 %) 1 (0.4 %) 0 (0.0 %) 1 (0.4 %)	22 15 (12.3 %) 1 (0.8 %) 1 (0.8 %) 3 (2.5 %) 0 (0.0 %) 3 (2.5 %) 1 (0.8 %) 0 (0.0 %) 3 (2.5 %) 2 (1.6 %) 1 (0.8 %) 0 (0.0 %) 2 (1.6 %) 0 (0.0 %) 0 (0.0 %) 2 (1.6 %) 2 (1.6 %) 3 (2.5 %) 0 (0.0 %) 0 (0.0 %)	– 0.065 1.000 0.340 0.693 – 0.693 0.340 0.550 1.000 – 1.000 0.550 1.000 – – 0.267 1.000 0.116 – 1.000

*FMC* first medical contact, *PCI* percutaneous coronary intervention, *CABG* coronary artery bypass graft, *DES* drug-eluting stent*, MI* myocardial infarction, *CVA* cerebrovascular accident, *TIMI* thrombolysis in myocardial infarction (bleeding as described by the TIMI bleeding criteria)

^*^Significant difference

^a^Including all procedures

^b^The number of patients with a pseudoaneurysm consists of patients treated with either surgery or if transfusion was necessitated

Procedural complications are also shown in Table 2. Cardiac arrest was the most common complication (2.1 %) followed by stent thrombosis (1.7 %). In 4 procedures (1.7 %) coronary dissection occurred. One patient (0.4 %) had coronary perforation with tamponade. One patient had tamponade most likely due to a temporary pacemaker lead. Before transport to Maastricht University Medical Centre for emergency cardiac surgery, this patient died as a result of rupture of the right ventricle. This was considered a procedure-related death. Procedural complications of patients receiving PPCI at Catharina Hospital are also shown in Table 2. There is no significant difference in procedural outcomes between these two groups.

### Follow-up

The follow-up period was 6 months. Ten patients were lost to follow-up. These were all foreigners who were transferred to a hospital in their home country for further rehabilitation. The follow-up data are shown in Table 3. During the first 30 days, 7 patients (3.2 %) died, of whom 5 (2.3 %) suffered a cardiac death. Two patients died a few minutes after the operator made the decision that continuing the procedure was no longer helpful. One patient died as a result of stent thrombosis after an elective multivessel PCI four days earlier in another hospital. This patient was unsuitable for CABG before the initial PCI. One patient died as a result of persisting cardiogenic shock after re-PCI for stent thrombosis. There was one procedure-related death as discussed earlier. There were 7 re-PCIs, 5 (2.4 %) were in the target vessel as a result of stent thrombosis.

**Table 3:** Cumulative follow-up

End point	VieCuri	Catharina	*p*
*0–30 days follow-up*			
Total^a^, n Lost to follow-up MACE total, *n* (%) Death – Cardiac, *n* (%) – Non-cardiac, *n* (%) MI, *n* (%) Re-PCI total, *n* (%) – Target lesion, *n* (%) – Target vessel, *n* (%) – Non-target vessel, *n* (%)	218 10 17 (7.9 %) 7 (3.2 %) 5 (2.3 %) 2 (0.9 %) 3 (1.4 %) 7 (3.2 %) 5 (2.4 %) 1 (0.5 %) 1 (0.5 %)	111 4 9 (8.1 %) 2 (1.8 %) 2 (1.8 %) 0 (0.0 %) 4 (3.6 %) 3 (2.7 %) 2 (1.8 %) 0 (0.0 %) 1 (0.9 %)	– – 0.797 0.723 1.000 0.546 0.232 1.000 1.000 1.000 1.000
*0–6 months follow-up*			
Total^a^, *n* MACE total, *n* (%) Death – Cardiac, *n* (%) – Non-cardiac, *n* (%) MI, *n* (%) Re-PCI total, *n* (%) – Target lesion, *n* (%) – Target vessel, *n* (%) – Non-target vessel, *n* (%) CABG emergency, *n* (%) CABG semi-elective, *n* (%) CVA, *n* (%) Ischaemic CVA, *n* (%) Major bleeding, *n* (%) Minor bleeding, *n* (%) Transfusion, *n* (%)	214 32 (15.0 %) 13 (6.1 %) 5 (2.4 %) 8 (3.7 %) 9 (4.2 %) 10 (4.7 %) 5 (2.3 %) 2 (0.9 %) 3 (1.4 %) 0 (0.0 %) 5 (2.3 %) 2 (0.9 %) 2 (0.9 %) 0 (0.0 %) 8 (3.7 %) 2 (0.9 %)	111 13 (11.7 %) 3 (2.7 %) 2 (1.8 %) 1 (0.9 %) 5 (4.5 %) 5 (4.5 %) 2 (1.8 %) 0 (0.0 %) 3 (2.7 %) 0 (0.0 %) 1 (0.9 %) 2 (1.8 %) 2 (1.8 %) 0 (0.0 %) 2 (1.8 %) 3 (2.7 %)	– 0.311 0.191 1.000 0.282 1.000 1.000 1.000 0.549 0.415 – 0.657 0.607 0.607 – 0.513 0.341

*MACE* major adverse cardiac events, *MI* myocardial infarction,* PCI* percutaneous coronary interventions, *CABG* coronary artery bypass graft, *CVA* cerebrovascular accident

^a^Patients with multiple procedures are counted as one

During the total follow-up period of 6 months, 13 patients (6.1 %) died. All cardiac deaths occurred in the first 30 days after the PPCI procedure. During the follow-up period of 6 months there was no significant difference in primary and secondary outcomes in patients receiving PPCI in VieCuri compared with Catharina Hospital.

## Discussion

This study presents procedural complications and clinical outcomes of a new off-site PPCI centre in the Netherlands. As shown in previous studies [3, 8, 14] our study confirms PPCI in STEMI patients at an off-site PCI centre to be safe and effective in the Netherlands. The percentage of emergency surgery in our study was 0.0 % which corresponds with the 0–1 % found in the literature. Patient characteristics and procedural specifications were similar in VieCuri and Catharina Hospital, although in the VieCuri group there were significantly more patients with Killip class II and the TIMI risk score was significantly higher.

In the Catharina Hospital group the system delay was significantly longer than in the VieCuri group. This is mainly due to a longer travel time. Furthermore, there were 21 patients who were first admitted to VieCuri before undergoing PPCI in Catharina Hospital, which will affect the time registration in a negative way. The occurrence of procedural complications was low in both groups. There was no significant difference in procedural complications between the two groups.

The study period in VieCuri was shorter (9 months) than in Catharina Hospital (12 months). Nevertheless, the number of patients in the VieCuri group was higher. There are a few explanations for this difference. First, part of this difference can be explained by adding the number of patients (*n* = 74) sent from non-PCI centres for PPCI in VieCuri. The Catharina Hospital group included only patients from VieCuri, and no patients from surrounding hospitals were included. Despite this, the difference in the number of patients remains high. Although the majority of STEMI patients were sent to Catharina Hospital, it is possible that some patients were sent to Maastricht University Medical Centre when Catharina Hospital was already occupied.

There is a significant difference in aspirin prescription, which can be corrected by the number of patients receiving a vitamin K antagonist due to atrial fibrillation. When no beta blocker was prescribed, a clear motivation was found in medical records.

The percentage of 30-day MACE was low in both the VieCuri and Catharina Hospital group at 7.9 and 8.1 %, respectively. Despite a longer reperfusion time in the Catharina Hospital group, there was no significant difference between the occurrence of MACE. This might be due to a reduced door-to-balloon time with a longer travel distance [24]. Moreover, VieCuri is a new PPCI centre. All cardiac deaths (2.3 %) occurred within 30 days for the VieCuri group. In the literature the percentage of in-hospital deaths of patients receiving PPCI in hospitals without surgical back-up varies from 4 to 9.8 % [10, 25]. In both trials the number of in-hospital deaths was significantly higher for the off-site PCI group. Occurrence of 30-day mortality in the study by Tomassini et al. [11] was 7.1 %. For the VieCuri group 30-day all-cause mortality was 3.2 % compared with 1.8 % in the Catharina Hospital group, which is not significant. In comparison with previously mentioned studies this percentage of total deaths is low. The occurrence of secondary outcomes is also very low in both study groups.

## Limitations

There are several limitations in our study. First, this is a retrospective study. Second, this study group is relatively small and providing a larger dataset would be preferred as this would stimulate the power of the study and gives a higher possibility to catch rare events. In this study the door-to-balloon time was not investigated, due to a difference in definition between VieCuri and Catharina Hospital. New studies should investigate whether, in geographically isolated areas, performing PPCI in experienced off-site PCI centres is superior to on-site PPCI due to a shorter reperfusion time and therefore decreased infarct size. In this study only STEMI patients were included. A study by IJkema et al. [26] shows that not all ECGs of patients with a transmural myocardial infarction have ST elevation. New studies should investigate the time to reperfusion and occurrence of MACE in this category too.

## Conclusion

This study reports the procedural and clinical outcomes in STEMI patients who underwent off-site PPCI at VieCuri Medical Centre. Results were compared with the results of STEMI patients who underwent on-site PPCI at Catharina Hospital. In both study groups the occurrence of procedural complications and MACE were low and no significant differences were found. The study results therefore confirm that the procedural and clinical outcomes of a new off-site intermediate-volume PPCI centre are comparable with those of an on-site high-volume PPCI centre.
